# Fusion Genes in Myeloid Malignancies

**DOI:** 10.3390/cancers16234055

**Published:** 2024-12-03

**Authors:** Chieh Hwee Ang, Hein Than, Tertius T. Tuy, Yeow Tee Goh

**Affiliations:** Department of Haematology, Singapore General Hospital, Singapore 169608, Singapore; hein.than@singhealth.com.sg (H.T.); tertius.tansloan.tuy@singhealth.com.sg (T.T.T.); goh.yeow.tee@singhealth.com.sg (Y.T.G.)

**Keywords:** fusion genes, myeloid malignancies, next-generation sequencing

## Abstract

Fusion genes arise from gross chromosomal rearrangements and have been closely linked to oncogenesis. In myeloid malignancies, fusion genes play an integral role in the establishment of diagnosis and prognostication. In the clinical management of patients with acute myeloid leukemia, fusion genes are deeply incorporated in risk stratification criteria to guide the choice of therapy. As a result of their intrinsic ability to define specific disease entities, oncogenic fusion genes also have immense potential to be developed as therapeutic targets and disease biomarkers. In the current era of genomic medicine, breakthroughs in innovation of sequencing techniques have led to a rise in the detection of novel fusion genes, and the concept of standard-of-care diagnostics continues to evolve in this field. In this review, we outline the molecular basis, mechanisms of action and clinical impact of fusion genes. We also discuss the pros and cons of available methodologies that can be used to detect fusion genes. To contextualize the challenges encountered in clinical practice pertaining to the diagnostic workup and management of myeloid malignancies with fusion genes, we share our experience and insights in the form of three clinical case studies.

## Simple Summary

Oncogenic fusion genes are instrumental in the pathogenesis, diagnosis, prognostication and treatment of myeloid malignancies. This review provides an overview of the clinical significance of fusion genes and discusses the strengths and limitations of available methods that can be used to detect these genes. Through a series of clinical cases, we highlight potential challenges that may be encountered in the management of myeloid malignancies with fusion genes.

## 1. Introduction

Myeloid malignancies encompass a broad spectrum of disease entities, which include myelodysplastic syndrome (MDS), myeloproliferative neoplasms (MPNs), and acute myeloid leukemia (AML). They are a group of vastly heterogeneous diseases which arise from genetic and epigenetic alterations, and are characterized by the clonal proliferation of abnormal hematopoietic progenitor cells [1,2,3]. Amongst the diverse range of acquired mutations with which myeloid malignancies are associated, chromosomal rearrangements that give rise to fusion genes represent an important class of somatic alterations that are pivotal in oncogenesis.

Fusion genes occur in approximately 30–40% of patients with AML [4,5,6] and 3% of MDS patients [7]. While the prevalence of fusion genes in *BCR::ABL1*-negative MPNs has not been well characterized, an increase in the frequency of acquired chromosomal aberrations has been observed in accelerated-phase MPNs and likely contributes to the pathogenesis of leukemic transformation [8].

Over the past decade, the topic of fusion genes in myeloid malignancies has garnered increasing attention in the community. Many of these chimeric genes are found to be oncogenic drivers, which are not only disease-defining and prognostic determinants, but also potential therapeutic targets and biomarkers of disease activity. In the field of MPNs, the discovery of *BCR::ABL1* fusion (arising from a reciprocal translocation between chromosomes 9 and 22), which is the hallmark of chronic myeloid leukemia (CML), is an archetypal example of how the diagnosis of a fusion gene has successfully transformed clinical outcomes through the development of effective targeted therapy [9]. With advances in state-of-the-art technologies such as high-throughput RNA sequencing, the number of detected fusion genes with pathogenic significance has grown exponentially. Outside of the realm of hematological malignancies, fusion genes have also been recognized as oncogenic drivers in solid-organ cancers [10].

This review outlines the molecular and clinical significance of fusion genes in myeloid malignancies and critically dissects the utility of current diagnostic modalities that can be employed to detect fusion genes. We conclude by sharing clinical insights and reflections based on real-world experience.

## 2. Mechanisms of Action of Fusion Genes

**Figure 1:**
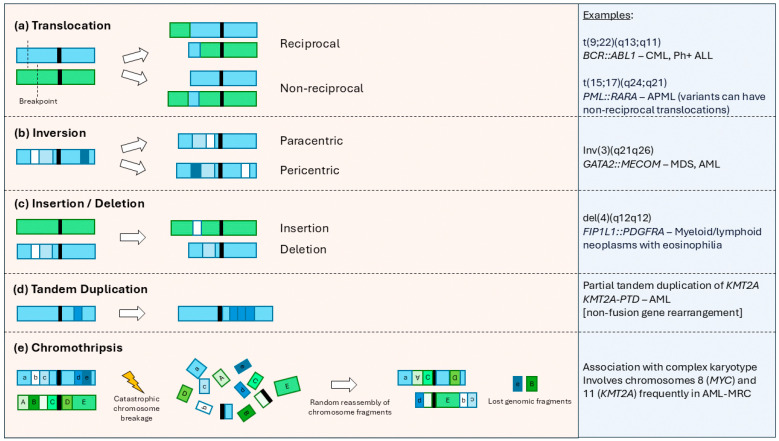
Structural chromosome rearrangements that can lead to the formation of fusion genes [11,12]. Examples of chromosomal aberrations with respective fusion gene products and clinical associations are listed in the panel on the right. Abbreviations: APML; acute promyelocytic leukemia, AML-MRC; acute myeloid leukemia with myelodysplasia-related changes, Ph+ ALL; Philadelphia-positive acute lymphoblastic leukemia.

Fusion genes can arise from chromosomal translocations, insertions, deletions, inversions, tandem duplication, and chromothripsis. Figure 1 illustrates the various mechanisms of chromosomal aberrations that can lead to the formation of fusion genes.

Fusion genes exert their oncogenic effects through one or more mechanisms, including the disruption of normal gene function, altered protein function, dysregulated protein expression, constitutive activation of signaling pathways, and epigenetic changes.

In the classical example of CML, the *BCR::ABL1* fusion gene encodes a multi-domain, constitutively active chimeric tyrosine kinase [13], in turn activating downstream signaling pathways that result in abnormal cellular adhesion, inhibition of apoptosis through inactivation of tumor suppressor genes [14,15], and eventually, uncontrolled proliferation of myeloid cells.

The orchestration of epigenetic alterations by fusion genes is well exemplified by the downstream molecular consequences of *KMT2A* fusion genes. By virtue of the role of KMT2A in histone methylation and transcriptional activation of *HOX* genes, KMT2A fusion proteins can induce broad changes in histone modification patterns, leading to aberrant gene expression profiles that favor leukemogenesis [16,17,18]. This is a recurrent theme that is similarly observed in AML with *RUNX1::RUNX1T1* fusion, as the resultant chimeric fusion proteins have also been shown to mediate the recruitment of chromatin-modifying enzymes to target genes [19].

## 3. Clinical Implications of Fusion Genes in Myeloid Malignancies

### 3.1. Establishment of Diagnosis

A strong association exists between specific fusion genes and particular subtypes of leukemia, resulting in genetically defined disease entities. The latest World Health Organization (WHO) classification [20] and International Consensus Classification (ICC) [21] have demonstrated unanimity in their retention of “AML with defining or recurrent genetic abnormalities” as a subcategory of AML, reflecting the essence of molecular classification in the diagnosis of AML (Table 1).

A prototypic example is acute promyelocytic leukemia (APML), a distinct subtype of AML that is defined by the presence of the *PML::RARA* fusion gene, which results from t(15;17)(q22;q12). Aside from its characteristic morphology, it is uniquely treated with a combination of all-trans-retinoic acid and arsenic trioxide with high response rates [22]. AML with *RUNX1::RUNX1T1* fusion represents another paradigm of how a single cytogenetic abnormality of t(8;21)(q22;q22) suffices in delineating a distinct leukemia subtype, in terms of morphology, immunophenotype, association with recurrent cooperating mutations, and favorable clinical outcomes.

AML classification aside, the detection of fusion genes supports the refinement of the diagnosis of rare disease subtypes, such as cases of undifferentiated hypereosinophilia with morphological findings that are not specific for a unifying diagnosis. This is especially relevant in the early phases of disease when the bone marrow shows only moderate histomorphologic changes [23].

### 3.2. Prognostication

In the prognostication of AML, cooperative groups such as the European Leukemia Net (ELN) have incorporated the presence of chromosomal aberrations and genetic mutations as part of their criteria for risk stratification (Table 2) [24]. This underscores the critical role that fusion genes play in facilitating risk-directed therapy.

### 3.3. Therapeutic Targets

Taking the molecular basis of fusion genes into account, fusion genes that encode tyrosine kinases, transcription factors, or proteins that interact with transcription factors and oncoproteins can give rise to the development of potential therapeutic targets [25]. Table 3 lists several targets that have been identified and translated into pharmacological interventions.

### 3.4. Monitoring of Disease Activity

Given that fusion genes may act as oncogenic drivers and are involved in canonical pathways in the pathogenesis of myeloid malignancies, they serve as surrogates of disease activity, and several (such as *CBFB::MYH11* and *RUNX1::RUNX1T1*) [24] have been identified as robust candidates of response biomarkers for monitoring of measurable residual disease (MRD).

## 4. Diagnostic Methods for the Detection of Fusion Genes

### 4.1. Karyotyping

Following the discovery of the Philadelphia chromosome in 1960 [38], conventional karyotyping has been adopted as a fundamental and routine investigation for the detection of chromosomal aberrations and structural variants in various hematological malignancies. Karyotyping involves culturing dividing cells, followed by the application of a mitosis inhibitor to arrest cells in metaphase so that chromosomes can be examined in a condensed form. Cells are then subjected to Giemsa banding and studied on slides with an image analyzer by a cytogeneticist. The resolution of karyotyping is approximately 5–7Mb of DNA [39], depending on the banding technique and the type and location of structural variant. The turnaround time is in the range of one to two weeks, contingent upon the complexity of the case and yield of metaphases.

This is a cost-effective but laborious method that is heavily reliant on the expertise of trained technicians and subjective analysis. In cases of complex cytogenetics, not all aberrations can be fully resolved by conventional karyotyping [40,41]. Recent advances in whole transcriptome sequencing revealed a high number of novel fusions in AML and MDS patients with complex cytogenetics [7], which may potentially be missed by conventional karyotyping. In addition, karyotyping is unable to detect cryptic translocations, which inherently involve chromosomal regions with similar G-banding patterns [42,43]. Fusion genes that lie close to telomeric ends of chromosomes also pose technical challenges for detection by conventional karyotyping [44].

### 4.2. Fluorescence In Situ Hybridization (FISH)

This is a technique that identifies chromosomal abnormalities with the use of fluorescently labeled DNA probes that hybridize complementary DNA of specified genetic sequences. Cells are then analyzed under fluorescence microscopy. FISH can be performed on interphase cells and does not require actively dividing cells. An apparent limitation of FISH is that it does not provide whole genome coverage and is a targeted assay that is only able to interrogate a single genomic region of interest. For genes with multiple fusion partners, such as *KMT2A* or *NUP98*, FISH can identify the gene rearrangement of interest but is unable to detect corresponding partner genes. To assess multiple aberrancies, several independent assays are required, which evokes additional cost and complexity in the assay. The turnaround time can be as fast as 24 to 48 h, which enables rapid confirmation of clinical diagnosis, such as in cases of suspected APML [45].

### 4.3. Optical Genome Mapping (OGM)

OGM, also referred to as next-generation cytogenomics, is a novel technique that maps optically imaged linearized DNA fragments to reference genome sequences. It is performed by the extraction of ultra-high molecular weight DNA, enzymatic labeling of DNA at specific motifs, followed by imaging of individual DNA molecules as they pass through a nanochannel flow cell. This method is centered upon DNA labeling as opposed to sequencing and directly visualizes DNA molecules in their native state, enabling rapid and efficient large-scale structural and copy number evaluation of the genome [46].

Potential drawbacks of this technique include the requirement for high-molecular-weight DNA isolated by specialized kits, and hence it cannot be performed on formalin-fixed paraffin-embedded (FFPE) tissues or on previously extracted DNA using conventional methods. OGM is also unable to detect variants located in centromeric or telomeric regions.

Recent studies have demonstrated that OGM has equal or better sensitivity compared to conventional karyotyping in the diagnostic workup of hematological malignancies [47,48,49]. In 2021, an international consortium for OGM in hematological malignancies was established for the creation of a consensus framework for the implementation of OGM in a clinical setting [50]. Though bioinformatic processing is provided by the manufacturer, one main challenge that was encountered with the use of this technology is the lack of harmonization of criteria for the reporting of clinical variants.

### 4.4. Polymerase Chain Reaction (PCR)

PCR is an enzymatic assay that allows for rapid amplification of a specific DNA fragment from a complex pool. Reverse transcriptase quantitative PCR (RT-qPCR) enables the detection and quantification of oncogenic fusion transcripts by transcribing RNA into complementary DNA, which is then used as the template for amplification. The quantitative nature of this assay facilitates monitoring of MRD status, which can guide clinical decisions.

RT-qPCR is a sensitive technique that can identify submicroscopic genetic aberrations with a limit of detection that can go as low as 1 target cell in 100,000 non-target cells. It also has a fast turnaround time of three to five days. However, the prerequisite is that it mandates prior knowledge of both fusion partners and a standard curve. As such, it is not capable of providing pan-genomic coverage, nor can it identify novel fusion genes.

Innovation in PCR methodology has led to the development of droplet digital PCR (ddPCR). This is an improvisation of digital PCR, which was first described in 1999 [51], but has only been increasingly adopted as a diagnostic tool in hematological malignancies in recent years [52]. In ddPCR, a sample is partitioned into several thousand droplets of individual PCR reactions to generate a limiting dilution, allowing for the quantification of target nucleic acids at single-molecule resolution and abrogating the need for a standard curve. The sensitivity of ddPCR has been demonstrated to be at least comparable to qPCR in the quantification of *JAK2 V617F* mutation in MPNs, with its ability to detect mutations at low allelic burdens [53]. This is a result of absolute quantification, which circumvents the difficulties of interpreting results when they fall below the limit of quantification or over the limit of detection [54]. Consequently, the field of molecular diagnostics has witnessed a sharp rise in the development of ddPCR assays for the detection of fusion genes.

### 4.5. Next-Generation Sequencing (NGS)

NGS refers to high-throughput and massively parallel sequencing, during which numerous small DNA fragments are sequenced and assembled [55]. Common NGS techniques employed to characterize the molecular landscape of malignant conditions include targeted gene panels, whole-genome sequencing, whole-exome sequencing and transcriptome sequencing (RNA-seq). Targeted gene panels, which are extensively employed in clinical laboratories, focus on established mutations and fusion genes recurrently described in myeloid malignancies. Whole-genome sequencing is performed de novo without prior knowledge of sequencing data, while exome sequencing refers to selective sequencing of coding regions of the genome after capturing of the exome. In contrast, RNA-seq interrogates regions of the genome that are transcribed and spliced into mature mRNA, providing information on alternative splicing, mutations, and changes in gene expression, facilitating an unbiased profiling of the target cells of interest.

The detection of fusion genes via DNA sequencing is challenging due to the need to cover a long intron region with many repeated sequences for precise identification of the fusion breakpoint. Also, complex transcriptional or post-transcriptional splicing processes can affect fusion gene detection at the DNA level [56]. As such, RNA-seq is recognized as a more suitable NGS-based approach for the detection of fusion genes.

Several groups have utilized RNA-seq to achieve unprecedented comprehensive profiling of somatic fusion genes in myeloid malignancies [2,7,57,58,59,60]. However, owing to the sheer enormity of the transcriptome, RNA-seq has poor sensitivity for the detection of fusion genes that are expressed at low levels or are diluted by the presence of accompanying non-malignant cell populations. Also, most non-recurrent fusion transcripts detected are stochastic events and are not pathogenic. While this is a powerful methodology, drawbacks associated with RNA-seq include the complexity of sequence library preparation and the need for a robust bioinformatics pipeline with integrated filtering strategies to ensure accurate analysis of results [61]. Though several algorithms for fusion gene selection have been described [62], to date there is no standardized way to interpret data from RNA-seq.

There is also variability with regard to practice and testing capabilities across different molecular laboratories. One major issue relates to the turnaround time of NGS results, which can range from one to three weeks, depending on the design and size of the panel, operational workflow, individual laboratory capability and resources.

### 4.6. Comparison of Diagnostic Methods

Table 4 summarizes the characteristics of currently available diagnostic methods for the detection of fusion genes.

## 5. Our Experience: Case Studies and Clinical Insights

In our institution, NGS has been established as an integral investigation in our diagnostic workflow for all patients presenting with suspected myeloid malignancies. We utilize a targeted and combined DNA and RNA panel that enables simultaneous detection of both DNA alterations and RNA fusions in a single run. Here, we share our experience in the form of three clinical vignettes that shed light on various real-world challenges and pitfalls in the detection of fusion genes. They also serve to illustrate the translational impact of fusion genes on clinical applications and therapeutic outcomes.

### 5.1. Detection of a Cryptic Translocation

A bone marrow aspirate from a 60-year-old lady presenting with skin rash and leukocytosis showed myeloblast infiltrate (25%), increased monocytic component, and dysgranulopoiesis. Interphase FISH using *MLL* breakapart probes revealed two fusion signals and one 5′*MLL* gene signal in 60% of 200 nuclei—suggesting that there was insertion of 5′*MLL* gene in a derivative chromosome. However, her karyotype demonstrated only trisomy 8, which provided no further insight into the underlying mechanism of *MLL* rearrangement.

She was treated with standard anthracycline-based ‘3+7’ induction chemotherapy, given clinical urgency in the setting of significant leukocytosis and extramedullary involvement. Subsequently, NGS returned positive for mutations in *U2AF1*, *ASXL1,* and *CBL* genes, together with *KMT2A::SORBS2* fusion. We were then able to clinch a comprehensive diagnosis of *KMT2A*-rearranged acute myelomonocytic leukemia, on a background of MDS. After induction chemotherapy, she achieved morphological remission but remained profoundly cytopenic. Given the presumptive diagnosis of underlying MDS, the decision was made to switch her consolidation therapy to azacitidine and venetoclax, as opposed to high-dose cytarabine. She was worked up for allogeneic stem cell transplantation but unfortunately succumbed to infective complications.

#### Clinical Pearls

This vignette showcases the utility of NGS in the detection of fusion genes in cases of cryptic translocations. Karyotyping was unable to detect the translocation between chromosomes 4 and 11, which gives rise to *KMT2A::SORBS2* fusion, a rare fusion that has been reported in pediatric AML [63,64]. The *KMT2A* gene, once termed a mixed-lineage leukemia (*MLL*) gene, is well known for its promiscuity, as it fuses with over 100 partner genes with numerous breakpoints in the context of hematological malignancies [64], giving rise to challenges in detection with conventional diagnostic methods. Through this case, we can appreciate the complementary roles of FISH and NGS in elucidating the mechanism of *KMT2A* rearrangement that underpins the pathophysiology of disease.

While our combined DNA and RNA NGS panel was useful in comprehensive molecular profiling, it had little impact on the choice of initial therapy due to the long turnaround time of two to three weeks. This highlights the importance of robust turnaround time for clinical NGS tests.

### 5.2. Unravelling a Potential Therapeutic Target

A 38-year-old man presented with generalized bone and joint pains and was found to have peripheral blood leukoerythroblastosis. His initial bone marrow findings were suggestive of *BCR::ABL1* fusion and *JAK2* mutation negative chronic MPN with fibrosis (reticulin score 4/4). He was started on hydroxyurea for cytoreduction, but deteriorated rapidly with fever and arthralgia and accelerated into leukemic phase (13% myeloblasts with an increased monocytic component comprising 6% promonocytes and 2% monoblasts) in his repeat bone marrow assessment three weeks later. Karyotyping showed complex cytogenetics with t(5;13)(q14;q12), t(10;18)(p15;q21), and t(11;19)(q23;p13.3). NGS panel detected *KMT2A::MLLT1* fusion. He was treated with cytarabine and venetoclax, and achieved hematological response with persistence of t(5;13). This prompted an exploratory RNA-seq analysis, which revealed a novel fusion *MEF2C::FLT3*, consistent with his karyotype t(5;13)(q14;q12). He was switched to maintenance therapy with gilteritinib and venetoclax, and was successfully bridged to allogeneic stem cell transplantation in an MRD-negative state. He remained in complete cytogenetic and molecular remission post-transplant with complete donor chimerism.

#### Clinical Pearls

This case highlights the challenges with a fibrotic marrow that can mask the findings of initial diagnostics, leading to false negative results and a potential delay in therapeutic management. Given the purported feasibility of the detection of fusion genes in bone marrow trephines [65,66], due consideration should be given to the option of performing FISH and NGS on FFPE sections of bone marrow trephine biopsies when encountering cases of fibrotic marrow with suboptimal or hemodiluted bone marrow aspirates, in order to optimize diagnostic yield.

*FLT3* fusion genes are a rare occurrence mostly described in cases of MPNs and myeloid/lymphoid neoplasms associated with eosinophilia [67,68]. The majority of cases with *FLT3* fusions exhibited a poor response to conventional chemotherapy, while patients who received a FLT3 inhibitor demonstrated at least a partial or transient response [69,70,71,72]. To our knowledge, *MEF2C::FLT3* fusion has not been previously reported in the literature. In our case, the identification of this novel fusion facilitated the use of a targeted FLT3 inhibitor (gilteritinib), which contributed to disease control while sparing excessive toxicity before transplant. The achievement of MRD negativity prior to allogeneic stem cell transplantation predicted the reduced risks of relapse post-transplant [73].

### 5.3. Resolution of a Diagnostic Dilemma and Impact on the Choice of Therapy

A 48-year-old lady with fever and severe abdominal pain was found to have 84% circulating blasts. Bone marrow aspiration demonstrated a leukemic infiltrate, which morphologically favored the diagnosis of AML without maturation. On immunophenotyping, blasts were found to express both myeloid and B-lymphoid markers including CD19 and CD22. Differentials entertained at that point included AML with aberrant B-lymphoid markers, or B/myeloid mixed phenotypic acute leukemia. As the patient was critically ill with concurrent infective complications and unfit for intensive chemotherapy, she was initially treated with a novel combination of inotuzumab ozogamicin, venetoclax, and dexamethasone, aimed at targeting both myeloblasts and CD22-expressing blasts. Subsequently, her karyotype showed trisomy 8 and interphase FISH was positive for *TP53* gene deletion at 17p13.1. *FLT3-ITD* PCR was also positive. No fusion genes were detected on the initial NGS panel. An additional RNA-seq study returned positive for *NUP98::NSD1* fusion.

She was then switched to azacitidine, venetoclax, and gilteritinib as she stabilized. Repeat bone marrow aspiration showed complete remission with flow cytometry demonstrating positive MRD with aberrant CD19+ expression. She was then treated with blinatumomab, a CD19/CD3 bispecific T-cell engager, as a bridge to allogeneic stem cell transplantation. She achieved complete molecular remission with complete donor chimerism following transplantation and was managed with prophylactic strategies to prevent disease relapse, including the use of donor lymphocyte infusions and maintenance with azacitidine in combination with a FLT3 inhibitor (sorafenib).

#### Clinical Pearls

*NUP98::NSD1* is a rare fusion that is more commonly associated with pediatric AML. In a comprehensive transcriptomic study of a large cohort of children and young adults with AML, 4.8% were found to have *NUP98::NSD1* fusion [74]. This fusion is recognized as one of the frequently underestimated fusion genes by conventional karyotyping [75], as both partner genes lie close to the telomeric ends of chromosomes 11 (*NUP98*) and 5 (*NSD1*) [76]. A high incidence of *FLT3-ITD* mutations has been reported in AML patients with *NUP98::NSD1* fusion, and it has been hypothesized that the interaction between *NUP98::NSD1* and *FLT3-ITD* confers a poor prognosis in this group [76,77].

In this case, the detection of *NUP98::NSD1* fusion enabled prompt diagnosis of a rare disease entity that portends an aggressive clinical course with poor outcomes. As *NUP98::NSD1* AML tends to respond poorly to conventional chemotherapy, there was greater impetus for the use of targeted therapy and immunotherapy for this patient. Regular monitoring of the fusion was practiced as its resurgence will enable the detection of an early molecular relapse and allow for pre-emptive intervention.

## 6. Future Directions

As RNA sequencing platforms become more widely accessible and affordable, they will be increasingly incorporated in routine diagnostics for hematological malignancies, refining our understanding of the role of fusion genes in pathogenesis. In an era of precision medicine, this will undoubtedly open new doors to novel therapeutic strategies. Genomic knowledge will also facilitate the repurposing of different chemotherapeutics in other cancers for their use in myeloid malignancies. One such example is the potential adaptation of TRK inhibitors, which were originally developed for the treatment of solid organ cancers expressing the *NTRK* fusion, as they may exhibit efficacy in rare cases of AML with *NTRK* fusion [78].

With greater utilization of NGS, concerted efforts towards the generation of a standardized and validated bioinformatics algorithm for the analysis of RNA-seq data are necessary in a bid to improve the clinical utility of this methodology. Artificial intelligence technologies may be the missing piece to this puzzle, given its powerful machine-learning capabilities and promising applications in annotation of sequencing data [79,80].

We foresee that with further advancements in sequencing approaches, techniques will also be geared towards a shorter turnaround time. Strategies that are currently being employed by molecular laboratories to achieve faster results include case prioritization and the design of optimized NGS panels [81]. Prompt identification of actionable fusion genes is especially relevant to the time-sensitive treatment of AML patients, since there is now an expanded spectrum of upfront novel treatment options potentially available. However, high costs associated with NGS-based approaches remain a practical issue that poses a conundrum in the clinical setting.

By nature of their cancer-specific expression, fusion genes serve as reliable markers of MRD and surrogates of disease activity. Given the importance of MRD monitoring in the management of myeloid malignancies (in particular AML) [82], the future will witness an increase in the development, validation, and availability of high-sensitivity assays (ddPCR and NGS-based) for monitoring fusion genes as MRD markers. This may eventually revolutionize the way we treat conventionally adverse-risk patients, especially if a deep molecular remission can be achieved with targeted therapy.

## 7. Conclusions

Fusion genes are paramount to the pathogenesis, diagnosis, and treatment of myeloid malignancies, with a profound impact on therapeutic approaches and clinical outcomes. With the advent of NGS, while we embrace its benefits and harness the prowess of RNA-seq in the detection of fusion genes, it is essential to recognize its limitations and shortfalls. To date, there is not a single diagnostic assay that can achieve high specificity and sensitivity of detection while being comprehensive (or targeted when necessary) and cost-effective with a rapid turnaround time. As such, there remains a place for conventional diagnostic methods, such as karyotyping, FISH, and PCR, in current clinical practice. Nevertheless, we believe that the standard of care in the diagnosis of myeloid malignancies will continue to evolve with the incorporation of pan-genomic platforms and improved access to novel technologies.

## Figures and Tables

**Table 1:** Leukemic fusion genes in the fifth edition of the World Health Organization (WHO) Classification of Hematolymphoid Tumors and International Consensus Classification (2022).

WHO (5th Edition)	ICC (2022)
*PML::RARA*	
*RUNX1::RUNX1T1*	
*CBFB::MYH11*	
*DEK::NUP214*	
RBM15::MRTFA *	
*BCR::ABL1*	
*KMT2A* rearrangement	*MLLT3::KMT2A* Other *KMT2A* rearrangement
*MECOM* rearrangement	*GATA2::MECOM(EVI1)* Other *MECOM* rearrangement
*NUP98* rearrangement	NUP98::NSD1 * NUP98::KDM5A * NUP98 and other partners *
AML with other defined genetic alterations	PRDM16::RPN1 * NPM1::MLF1 * KAT6A::CREBBP * ETV6::MNX * PICALM::MLLT10 * FUS::ERG * RUNX1::CBFA2T3 * CBFA2T3::GLIS2 *

Fusion genes listed under WHO classification belong to the category of “AML with defining genetic abnormalities”. Fusion genes listed under ICC are categorized under “AML with recurrent genetic abnormalities”, aside from those marked with *, which are classified under “AML with other rare recurring translocations”.

**Table 2:** Leukemic fusion genes incorporated in European Leukemia Net (ELN) 2022 risk stratification criteria for acute myeloid leukemia.

Risk Category	ELN 2022
Favorable	t(8;21)(q22;q22.1); *RUNX1::RUNX1T1* inv(16)(p13.1;q22) or t(16;16)(p13.1;q22); *CBFB::MYH11*
Intermediate	t(9;11)(p21.3;q23.3); *MLLT3::KMT2A*
Adverse	t(6;9)(p23;q34.1); *DEK::NUP214* t(v;11q23.3); *KMT2A* rearranged t(9;22)(q34.1;q11.2); *BCR::ABL1* t(8;16)(p11.2;p13.3); *KAT6A::CREBBP* inv(3)(q21.3;q26.2) or t(3;3)(q21.3;q26.2); *GATA2::MECOM (EVI1)*

**Table 3:** Examples of targeted therapy in myeloid malignancies with fusion genes.

Fusion Gene(s) of Interest	Target Category	Drug	Mechanism of Action
*BCR::ABL1* *FIP1L1::PDGFRA*	Tyrosine kinase	Tyrosine kinase inhibitors (targets) [26] ○ Imatinib (BCR-ABL, PDGFR, KIT) ○ Dasatinib (BCR-ABL, PDGFR, KIT, VEGFR, SRC) ○ Nilotinib (BCR-ABL, PDGFR, KIT, VEGF) ○ Bosutinib (BCR-ABL, SRC) ○ Ponatinib (BCR-ABL, PDGFR, KIT)	Inhibition of constitutive activation of tyrosine kinase activity prevents transmission of proliferative signals to the nucleus and induces apoptosis of malignant cells [27].
*PML::RARA*	Transcription factors	All-trans-retinoic acid (ATRA) [28]	In the absence of retinoic acid, RARA represses the transcription of target genes involved in myeloid differentiation. ATRA binds PML-RARA and converts it to an effective transcriptional activator, inducing differentiation [29].
*CBFB::MYH11*	Downstream protein-protein interactions	AI-10-49 [30]	Inhibition of protein-protein interactions of CBFB-MYH11 with RUNX1 restores RUNX1 transcriptional activity and prevents recruitment of DNMT3A to target genes [31].
*KMT2A* rearranged fusion genes	Complexes associated with transcription factors	Menin inhibitors [32] ○ Revumenib [33] ○ Ziftomenib [34]	KMT2A fusion interacts directly with tumor suppressor Menin and leads to hypermethylation and aberrant gene expression including upregulation of *HOX* gene expression, enhanced proliferation, and blocking differentiation [35]. Disruption of KMT2A-menin complex inhibits leukemogenic transcriptional activity.
*DEK::NUP214* *NUP98::NSD1*	Oncoprotein interactors	Chromosome region maintenance 1; exportin 1 (CRM1) inhibitor ○ Selinexor [36]	Nucleophorin fusions inhibit CRM1-mediated nuclear export of substrates, resulting in aberrant transcriptional activity from the accumulation of CRM1-regulated transcription factors [37]. CRM1 inhibitors disrupt protein complexes formed by nucleophorin fusions with other transcription factors.

**Table 4:** Characteristics of diagnostic methods for the detection of fusion genes.

	Karyotyping	FISH	PCR	OGM	NGS
Relative specificity	++	+++	+++	+++	+++
Relative sensitivity	+	++	+++	+++	++
Turnaround time	1 to 2 weeks	1 to 2 days	3 to 5 days	3 to 5 days	1 to 3 weeks
Sample requirements	Actively dividing cells, cell culture	Can be performed on interphase cells and FFPE		Ultra-high molecular weight DNA isolated by specialized kits	DNA or RNA isolated by conventional kits
Interrogation approach	Unbiased	Need for pre-defined targets		Unbiased	Unbiased (Option of pre-defined targets in targeted NGS panels)
Demands for logistics and manpower	Trained cytogeneticist		Trained lab scientist	Laboratory infrastructure Bioinformatics pipeline	
Cost	Low	Moderate	Moderate	High	High
Blind spots	Cryptic structural variants	Unable to identify novel fusion partners		Variant reporting not standardized—detection of variants of undetermined significance	

A subjective score is assigned to the relative specificity and sensitivity of each diagnostic method, denoted by the number of ‘+’ symbols.
